# Biosynthesized zinc oxide nanoparticles from *Cycas revoluta* seed extract demonstrate significant wound healing, antimicrobial, antioxidant, and cytotoxic potential

**DOI:** 10.3389/fphar.2025.1605717

**Published:** 2025-09-25

**Authors:** Hajar Hassan AlWadai, Shifhali Morabad, Uday M. Muddapur, Abdulrahman Manaa Alamri, Bader Aldoah, Adel Moalwi, Nasser A. N. Alzerwi, Saeed Ali Alsareii, Mater H. Mahnashi, Ibrahim Ahmed Shaikh, Aejaz Abdullatif Khan, Basheerahmed Abdulaziz Mannasaheb

**Affiliations:** ^1^ Department of Surgery, College of Medicine, Najran University, Najran, Saudi Arabia; ^2^ Department of Biotechnology, BVB Campus, KLE Technological University, Hubballi, Karnataka, India; ^3^ Department of Surgery, College of Medicine, Majmaah University, Ministry of Education, Al-Majmaah, Saudi Arabia; ^4^ Department of Pharmaceutical Chemistry, College of Pharmacy, King Khalid University, Abha, Saudi Arabia; ^5^ Department of Pharmacology, College of Pharmacy, Najran University, Najran, Saudi Arabia; ^6^ Department of General Science, Ibn Sina National College for Medical Studies, Jeddah, Saudi Arabia; ^7^ Department of Pharmacy Practice, College of Pharmacy, AlMaarefa University, Riyadh, Saudi Arabia

**Keywords:** zinc oxide nanoparticles, *Cycas revoluta*, wound healing, antibacterial, green synthesis, anticancer

## Abstract

**Introduction:**

Nanotechnology is an innovative approach that involves the development, production, and application of materials via altering their shape and size on the nanometer scale, which ranges from 1 to 100 nm. Zinc Oxide Nanoparticles (ZnONPs) are metal-oxide nanomaterials with distinct physical and chemical properties that make them valuable and adaptable inorganic compounds.

**Methods:**

The *C. revoluta* extract was subjected to comprehensive phytochemical analysis, and showed presence of triterpenoids, alkaloids, saponins, steroids, resins, diterpenes, and coumarins. The ZnONPs synthesized using *C. revoluta* extract were optimized by synthesizing zinc nanoparticles at different temperatures and pH. The synthesis of ZnONPs was determined by the UV–Vis spectrophotometry, with the absorbed peak being observed at 364 nm. The characterization of ZnONPs was performed with XRD, FTIR, SEM, TEM, and EDX analysis.

**Results:**

The surface morphology of the ZnONPs was analyzed using SEM, which revealed their spherical nature and particle size was determined to be 30 nm. Significant peaks were observed in the XRD pattern, confirming their sphere-like structure. FTIR spectra were recorded to determine the groups of biomolecules involved in synthesis. ZnONPs’ antibacterial efficacy against gram + ve and gram -ve bacteria, were positive. The ZnONPs demonstrated significant cytotoxic activity against TNBC MDAMB-231 cell line with an IC_50_ of 58.39 μg/mL. The percentage DPPH radical scavenging activity was 72.36% at 100 μg/mL concentration. Furthermore, ZnONPs showed significant (p < 0.001) wound healing characteristics in the excision wound model in rats with 99.29% wound contraction on treatment Day 16.

**Discussion:**

The ZnONPs showed increased cell migration potential in the in vitro scratch assay, highlighting their potential for wound care and tissue regeneration applications. In future, more in-depth research and clinical evaluation are warranted to fully explore the therapeutic potential of these environmentally friendly ZnONPs.

## 1 Introduction

Nanotechnology has become a widely recognized cutting-edge technology, with various applications in sectors such as food processing, mechanical engineering, chemicals, and pharmaceuticals. A key step in this field is the development of nanoscale materials using various metals (Anandharamakrishnan, 2014). These materials typically consist of single-dimensional particles with a minimum dimension of 100 nm (nm), and even zero-dimensional particles such as quantum dots (Kościk et al., 2021). Metal nanoparticles have special physical and chemical characteristics. Zinc oxide (ZnO) has been identified as one of the most advantageous metal oxides for multifunctional applications and can be utilized at the nanoscale level because of its unique properties in terms of electron mobility, optical electron mobility, semi-conductance, transparency, and biomedical properties. The distinct optical, electrical, catalytic, and magnetic properties make them interesting and versatile (Singhal et al., 2011). It is relatively easy to modify the physical and chemical behaviors of ZnO nanoparticles (ZnONPs) by varying the morphology of the nanoparticles through different synthesis pathways, intermediates, or materials (Bala et al., 2015).

ZnONPs are utilized in various biomedical engineering applications, such as tissue regeneration and implant coatings, as well as bio-imaging and wound healing. Owing to its benefits, ZnO has been extensively used as a photocatalyst, along with semiconductors, to remove organic contaminants from wastewater (Akl et al., 2012; Hassan et al., 2015). ZnONPs and colloidal suspensions with a high efficiency for the photodegradation of organic compounds are being leveraged for prophylactic (preventative) applications, environmental cleanup, and self-cleaning paints (Antoine et al., 2012). The nanoparticles in the paint react with pollutants when exposed to UV light, thus eliminating them from the surrounding air and preventing the buildup of discoloration.

Researchers are increasingly turning to green synthesis for manufacturing nanoparticles for pharmaceutical applications as the demand for environmentally friendly nanoparticles is on the rise (Akl et al., 2012). However, synthesis through chemical approaches such as micelle chemical precipitation, sol-gel process, hydrothermal method pyrolysis, and chemical vapor deposition causes hazardous chemical species to adsorb on the surface, potentially having detrimental effects on medical applications and the environment as well. The synthesis and stabilization of nanoparticles involve chemicals that are known to be hazardous and generate undesirable byproducts that are incompatible with the environment (Singhal et al., 2011). Plant-based nanoparticle synthesis is becoming increasingly popular because it is easier, faster, and cheaper.

The gymnosperm plant *C*. *revoluta*, also referred to as the sago palm, is a member of the Cycadaceae family. Cycas species are indigenous to Asia and can be found in the west in India and Sri Lanka, the northeast in China and Japan, and the south-east in Asia and Indonesia (Deora and Shekhawat, 2020). This symmetrical plant is supported by a crown of glossy dark green leaves. The trunk of the plant is thick with a diameter of approximately 20–30 cm. At reproductive maturity, the leaves measure 50–150 cm (20–59 cm) in length and have a deep, semi-glossy green color. Historically, the consumption of sago palms has been linked to several treatments including blood pressure management, skin condition improvement, relief from hematemesis, alleviation of gastrointestinal distress, hypertension management, cough suppression, and promotion of hair growth (Deora and Shekhawat, 2020). The natural extracts of *C*. *revoluta* have many medicinal properties. It inhibits gastric cancer cell proliferation and potentiates anti-cancer activity (Cui et al., 2019). Terminal shoots are used as anticoagulant diuretics (Ponsonby et al., 1987). Tender leaves are edible and the leaves juice is used to manage flatulence and vomiting (Chopra et al., 1986). C. *revoluta* leaf tincture contains cytochrome P450 aromatase inhibitors, and are being studied for the management of estrogen-dependent cancers (Kowalska et al., 1995). It is rich in amino acids, terpenes, diterpenoids, triterpenoids, sterols, esters, flavonoids, glycosides, fatty acids, benzenoids, and steroids. The synthesis of ZnONPs from C. *revoluta* for its therapeutic potential have not been studied previously.

**FIGURE 1:**
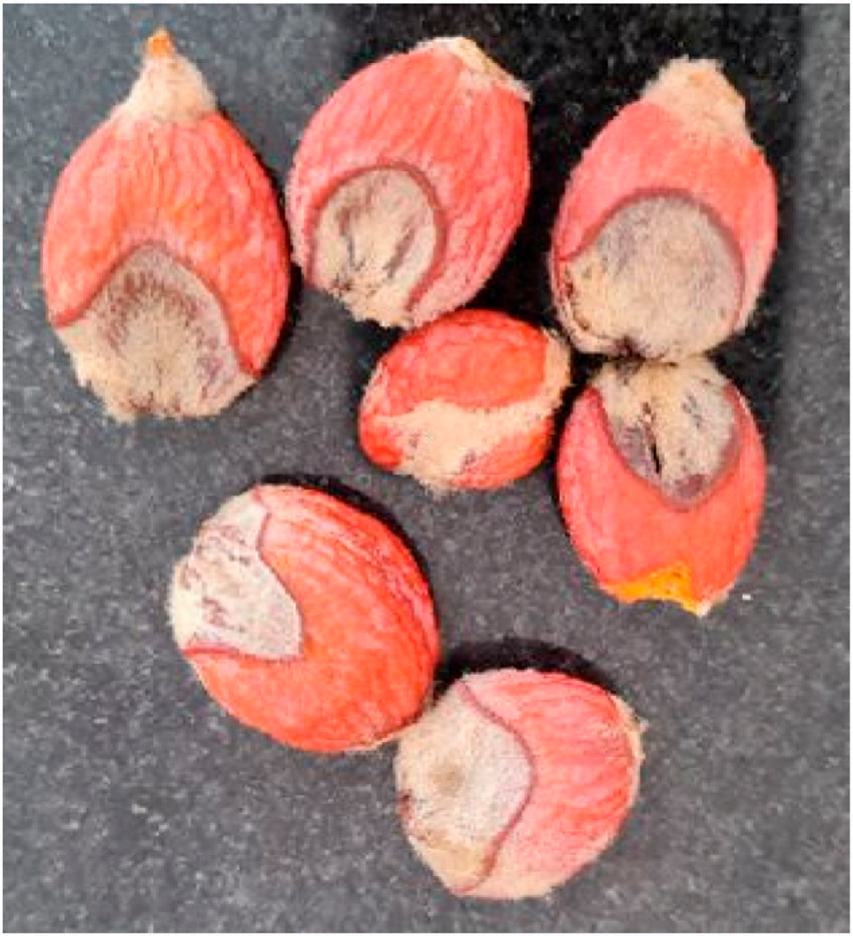
The seeds of *Cycas revoluta* plant.

In the current study, the ZnONPs were synthesized using a green synthesis method using *Cycas revoluta* seed extract (Figure 1). This method uses the stabilizing and reducing agents found in the seed extract to help fabricate biocompatible ZnONPs. The green synthesis method not only uses fewer harmful chemicals, but it also makes the nanoparticles safer, which renders them ideal for biomedical uses like treating cancer and healing wounds. Biogenic ZnONPs have special physical and chemical properties that can be changed to fit certain medical needs. The synthesized ZnONPs were further tested for their antimicrobial, antioxidant, wound healing, and cytotoxic potential. The results show that *C. revoluta*-mediated ZnONPs could be used as multifunctional agents in biomedical applications because they are more biocompatible and effective than nanoparticles synthesized by chemical methods.

### 1.1 Novelty of the study

Although the wound healing potential of natural products and ZnONPs has been extensively documented over the past decades (Criollo-Mendoza et al., 2023; Selim et al., 2024; Khan et al., 2021), the present study distinguishes itself by employing a green synthesis approach using *C. revoluta* seed extract, a plant source not previously explored for nanoparticle fabrication. The unique phytochemical profile of *C. revoluta*, rich in triterpenoids, diterpenes, and coumarins, may impart distinct surface chemistry and biological activity to the ZnONPs. This study not only optimizes synthesis parameters (temperature and pH) but also integrates multi-modal characterization (UV–Vis, XRD, FTIR, SEM, EDX) and evaluates cytotoxicity against TNBC MDAMB-231 cells, alongside *in vivo* wound healing and *in vitro* scratch assays. These combined assessments provide a comprehensive therapeutic profile, highlighting the potential of *C. revoluta*-mediated ZnONPs as a biocompatible, multifunctional agent for wound care and cancer therapy. Thus, the novelty lies in the plant source, synthesis optimization, and dual therapeutic evaluation, which collectively advance the current understanding of ZnONPs applications.

## 2 Methodology

### 2.1 Collection of plant and extraction

*C*. *revoluta* seeds were collected in the Hubballi KLE Campus, India. The extract was prepared by washing the seeds with distilled water. The seeds were shade-dried, then 50 g of them were coarsely chopped and mashed with a mortar and pestle. In a sterile beaker, the seed powder was mixed with 500 mL of distilled water and cooked for 15 min on a Bunsen burner. Whatman filter paper was used to filter the extract once it had cooled. After 15 min, the filtrate was centrifuged at 6,000 rpm. The supernatant was stored at −20 ° for future use.

### 2.2 Pharmacognostic screening of phytocompounds

In the current study, the aqueous extract was screened for the presence of phytochemical by the method described previously (Trease and Evans, 1989; Baliyan et al., 2022; Gaire et al., 2011; De et al., 2010; Sunil et al., 2012; Mace et al., 1976; Kumar et al., 2007; Parekh and Chanda, 2010; Harborne and Williams, 2010; Edeoga et al., 2005).

### 2.3 Zinc oxide nanoparticle synthesis

Zinc acetate solution (0.1 M) was made by dissolving 2.195 g of zinc acetate in 100 mL distillate water. The solution was magnetically agitated for an hour to completely dissolve the zinc acetate. The extract (20 mL) was added drop by drop to an 80 mL zinc acetate solution and stirred for 1 hour. The pH was adjusted to 10 by adding 2M NaOH and heated at 50 °C for 1 hour in a water bath. The solution was agitated for 2 h with a magnetic stirrer. A white crystalline precipitate was developed and washed multiple times with distilled water while the pH was kept at 7. A UV-1800 spectrophotometer was used to evaluate the precipitate’s absorbance in the 200–800 nm wavelength range in order to confirm the ZnONPs in the sample. The precipitate was dried in a hot air oven at 50 °C and stored in powder form for further use.

### 2.4 Effect of pH

This study examined the impact of pH in a variety of situations, including alkaline, neutral, and acidic ones. Following the dropwise addition of the extract to the zinc acetate solution, the pH was adjusted using NaOH to achieve the desired conditions for nanoparticle formation. HCl and NaOH were included to keep the pH at a range of 6, 8, and 11. The absorbance of the samples was measured using a UV-Vis spectrophotometer.

### 2.5 Effect of temperature

The formation of ZnONPs was influenced by the temperature. For the temperature optimization study, the heating step was modified to 30 °C, 40 °C, and 60 °C. Each batch was subjected to identical stirring and pH conditions, with only the temperature being varied to assess its influence on nanoparticle formation and optical properties. UV spectra were obtained for the aqueous colloidal suspensions at different temperature ranges.

### 2.6 Characterization and analysis of ZnONPs

Nanoparticle characterization can be achieved using two different methods. The first method involves analyzing physical properties, including particle size, shape, monodispersity, and crystal structure. The second method focuses on examining the chemical properties of the particles, such as the presence of ligands and bonded conjugated molecules. Different techniques, including as XRD, SEM-EDS, FTIR, and UV-vis spectroscopy, were employed to analyze the ZnONPs (Koczkur et al., 2015; Eid, 2022; Anandalakshmi et al., 2016).

#### 2.6.1 UV-vis spectrophotometry

Ten milliliters of distilled water was used to dissolve 10 mg of the ZnONPs. The absorbance spectra were measured using an optical UV-4000 UV-Vis spectrophotometer (Germany) with a wavelength range of 200–800 nm. The baseline was established using distill water as the blank. A graph of absorption vs. wavelength was used to identify the wavelength with the highest absorbance.

#### 2.6.2 SEM-EDS analysis

Data regarding the exterior morphological traits, construction, chemical content, and orientation of the material being investigated were obtained using SEM-EDS (manufactured JEOL model number JSM-IT500L). An electron beam was used to examine the samples after they had been dehydrated, which resulted in the acquisition of images and size information of the ZnONPs (Shaban et al., 2023; Mansour, 2017).

#### 2.6.3 Transmission electron microscopy (TEM) analysis

To conduct TEM examination, ZnONPs synthesized through green synthesis method were placed on carbon-coated copper grids and allowed to evaporate (Labulo et al., 2022). TEM measurements were performed using a JEOL JEM-2100F-UHR field-emission transmission electron microscope equipped with a 1k-CCD camera and a Gatan GIF 2001 energy filter. The instrument, manufactured by JEOL (Japan), operated at an accelerating voltage of 80 kV.

#### 2.6.4 FTIR

A Nicolet 5700 FTIR Spectrometer was used to perform infrared spectroscopy using Fourier transform (FTIR) to examine the range of wavelengths that show the chemical relationships and possible groups with useful properties found in ZnONPs. The materials were pressed together, ground into pellets, and analyzed using the potassium bromide (KBr) method. The spectral range was between 4,000 and 400 cm−1, and the accuracy was fixed at 1 cm−1 (Eid, 2022; Shojaei and Azhari, 2018).

#### 2.6.5 X-ray diffraction (XRD)

A PAN analytical Xpert Pro diffractometer with Cu Kα radiation was used for XRD experiments to determine the crystal structure. The scan speed was set to 8° min^−1^ and the range was 20°–80° (Fultz et al., 2013).

### 2.7 Biological activity

#### 2.7.1 Antioxidant activity DPPH assay

Different concentrations of ZnONPs were prepared separately in the test tubes. One milliliter of DPPH solution in methanol was added to the reaction mixture, which was then agitated briskly and allowed to stand at room temperature in a dark room for half an hour. A UV Visible spectrophotometer set to 517 nm was used to measure absorbance (Bagewadi et al., 2019; Baliyan et al., 2022).

$$\%\text{ DPPH Scavenging assay }\left(\%\right)=\frac{\left(\text{abs}\;\text{control}-\text{abs}\;\text{sample}\right)\times 100}{\left(\text{abs}\;\text{control}\right)}$$

#### 2.7.2 Antimicrobial assay

Antimicrobial assays of different plant extracts were performed using the agar disc diffusion method on nutrient agar plates (Balouiri et al., 2016). Antimicrobial activity was tested against five pathogens: gram (−) bacteria included *E. col*, *P. aeruginosa*, *Z*. *mobilis*; gram (+) bacteria included *S. aureus and B. subtilis*. Bacterial cultures were sub-cultured from glycerol stocks and grown in a nutrient broth for 24 h. Nutrient agar plates were used for the analysis. Each bacterium was uniformly spread on agar plates. Paper discs (Whatman) were soaked in extract, zinc acetate, ZnONPs, and standard (ampicillin) respectively for 3–4 h, and loaded onto agar plates, and were incubated at 37 °C for 24 h and zone of inhibition were determined (Horváth et al., 2015). The size of the inhibition zone is a measure of the efficacy of the plant extracts or chemicals against microbes (Balouiri et al., 2016; Ribut et al., 2018).

#### 2.7.3 Antifungal activity (disc diffusion)

The activity was checked on potato dextrose agar plates. It was tested against the fungal strain *Candida albicans* (Lab grown strain). The organisms were sub-cultured overnight in a broth containing PDB by adding loopful stock culture. Potato dextrose agar plates were prepared and allowed to solidify. Paper discs (Whatman) were soaked in zinc acetate solution, plant extracts, ZnONPs, and standard fluconazole solution for 3–4 h (Horváth et al., 2015).

#### 2.7.4 Anticancer activity against breast cancer cells (MDAMB-231)

This study investigated the effect of ZnONPs synthesized from *C. revoluta* on the viability of breast cancer cells (MDA-MB-231) using an MTT colorimetric assay (Carmichael et al., 1987).

#### 2.7.5 *In vitro* wound healing test

This study examined how ZnONPs affected the migratory characteristics of L929 cells. Initially, L929 cells were cultivated in 12-well plates using DMEM supplemented with 10% FBS and 2% penicillin-streptomycin. Once the cells had confluently formed a consolidated monolayer with a density of around 50,000 cells per well, a scratch was made in the cell layer using a sterile 100 μL pipette tip. The cells were grown at 37 °C with 5% CO_2_ for 24 h. MagVision software at 4X resolution was used to quantitatively assess the gap space in mm. The rates of migration and wound closure were computed as per standard procedure (Moalwi et al., 2024).

#### 2.7.6 *In vivo* assessment

##### 2.7.6.1 Animals and grouping

The *in vivo* excision wound model was conducted on Wistar rats of both sexes, weighing around 200 g. The animals were kept roughly 20 °C–22 °C in a controlled setting, where key external factors such as temperature, humidity, light-dark cycles (12 h), noise levels, and cage conditions were carefully regulated and monitored to minimize variability in experimental outcomes. Their dwelling cages consisted of husk-lined polypropylene cages that were replaced every 24 h. The rats were given free access to water and a standard pellet diet. Following approval by the Scientific and Ethical Committee of Najran University, the animals were divided into three groups randomly with six animals (n = 6) in each group as follows: Group I: Animals receiving no therapy (only ointment base). Group II: Animals receiving ZnONPs ointment. Group III: Animals receiving standard povidone-iodine ointment.

##### 2.7.6.2 Preparation of ointment

A British Pharmacopoeia formula was used to formulate the ointment (Alsareii et al., 2023; Alsareii et al., 2022). The contents included cetostearyl alcohol, white soft paraffin, wool fat, and hard paraffin. The ingredients were added in a specific order, considering the melting point of each ingredient. 5 g of cetostearyl alcohol, 85g paraffin, and wool fat were added to obtain 100 g of ointment base. The ingredients were dissolved and continuously mixed until homogenous consistency was achieved. After the heat was turned off, the liquid swirled until it reached the room temperature. Five grams of *C. revoluta* ZnONPs was combined with a portion of the basic ointment base to create an ointment of 5% (w/w). The ointment was used externally to the lesions/wounds for 21 days during the course of the treatment.

##### 2.7.6.3 *In vivo* wound healing excision model

Anesthetized rats were operated on according to the excision wound model protocol (Alsareii et al., 2022). The animals received 80 mg/kg ketamine and 5 mg/kg diazepam (i.p) (AVMA, 2020). On the shaved upper back, a 23 ± 1 mm^2^ circular wound was carefully made at an average depth of 2 mm. As specified in the grouping and dosage section, ZnONPs-containing ointment and medication-free ointment were administered topically daily. Injury was initiated on day 0. The percentage of wound contraction was calculated after the wound healing process was monitored at several points in time, noting the wound’s progressive closure and the development of new epithelial tissue.

##### 2.7.6.4 Estimation of hydroxyproline content

The amount of hydroxyproline in the tissues from the excised tissue was determined. A hot oven (60 °C) was used to dehydrate the tissue samples. The materials were next hydrolyzed using a strong hydrochloric acid (6N HCl; pH ≈ 1) solution for 4 h at a noticeably higher temperature of 130 °C. After the hydrolysates were adjusted to pH 7, oxidation was carried out for 20 min using chloramine-T. Following meticulous agitation, the samples were examined using an ultraviolet spectrophotometer set to 557 nm as the wavelength (Alsareii et al., 2023).

##### 2.7.6.5 Histopathology

The animals were sacrificed after being given ketamine (80 mg/kg, i. p.) and diazepam (5 mg/kg i. p) to produce anesthesia, and the animals were euthanized using cervical dislocation method (AVMA, 2020). Samples of the healed wound tissue from all groups were taken after the healing period post-excisional wounding. The samples were fixed for histological tissue analysis using 10% formalin. The tissue samples were stained with eosin and hematoxylin and examined under a light microscope. Van Gieson staining targeted collagen fibers in skin tissue. The tissue collagen levels were measured under a microscope (Alsareii et al., 2022).

### 2.8 Statistical

All data were statistically analyzed using GraphPad Prism software, version 6.01 (GraphPad Software Inc., San Diego, California, United States). One-way analysis of variance (ANOVA) was employed to assess differences among multiple experimental groups. To control for the risk of type I error arising from repeated pairwise comparisons, Tukey’s *post hoc* test was applied following ANOVA for intergroup comparisons. Data were expressed as mean ± standard deviation (SD), and a p-value of less than 0.05 was considered statistically significant.

## 3 Results and discussion

### 3.1 Phytochemical qualitative analysis

The phytochemical profile of *C. revoluta* seed extract showed the presence of various phytocompounds, including alkaloids, saponins, triterpenoids, steroids, resins, diterpenes, and coumarins (Table 1). These phytochemicals have potent reducing and capping properties that can facilitate the synthesis of stable, eco-friendly metal nanoparticles. This rich phytochemical profile suggests immense potential for the development of green synthesis techniques and various biomedical applications.

**TABLE 1:** Phytochemicals present in the *C. revoluta* seed extract.

S. No	Phytochemical	Inference
1	Alkaloid	Present
2	Flavonoid	Absent
3	Saponin	Present
4	Steroids	Present
5	Triterpenoids	Present
6	Tannin	Absent
7	Volatile oil	Absent
8	Resins	Present
9	Anthocyanosides	Absent
10	Catechin	Absent
11	Diterpenes	Present
12	Coumarins	Present
13	Anthraquinones	Absent
14	Phenols	Absent
15	Glycosides	Absent

### 3.2 Optimization of pH for synthesis of ZnONPs

**FIGURE 2:**
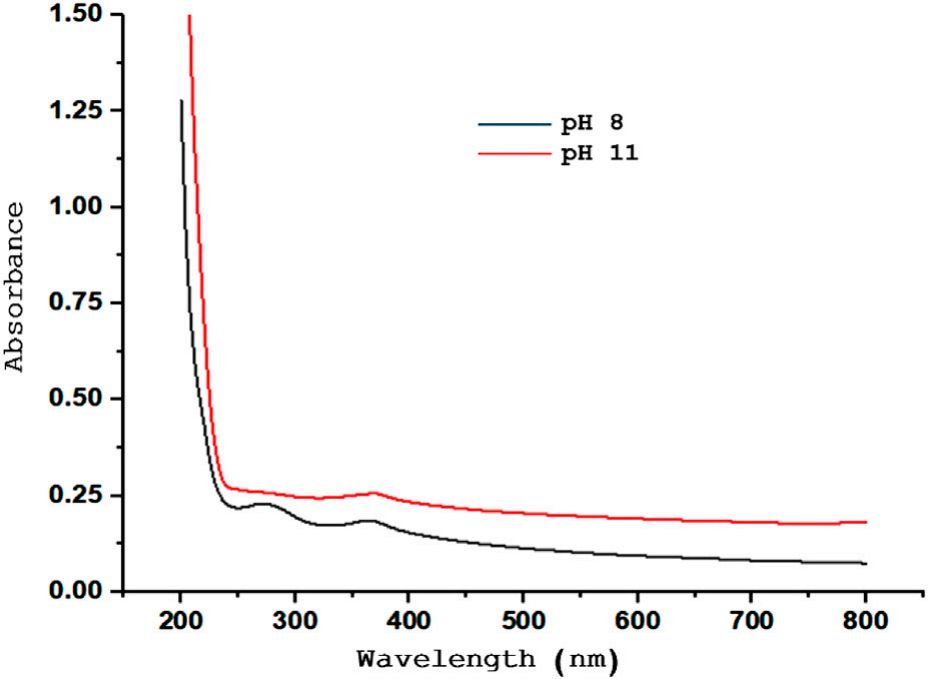
Optimization of pH for the synthesis of ZnONPs.

The effects of the pH were examined under different conditions. Figure 2 illustrates the impact of pH changes on the UV-Vis spectra for ZnONPs synthesized at pH 8, and pH 11. The optical properties were also confirmed by UV–Vis analysis with an optical band gap at 3.29 (3.31), 3.33 (3.31) and 3.31 (3.29) eV corresponding to ZnONPs. The spectra of ZnONPs depicts an emission band in the UV range at pH 11 (Ribut et al., 2018). The optimum pH for the synthesis was at pH 10 which gave the highest yield of ZnONPs.

### 3.3 Optimization of temperature for synthesis of ZnONPs

**FIGURE 3:**
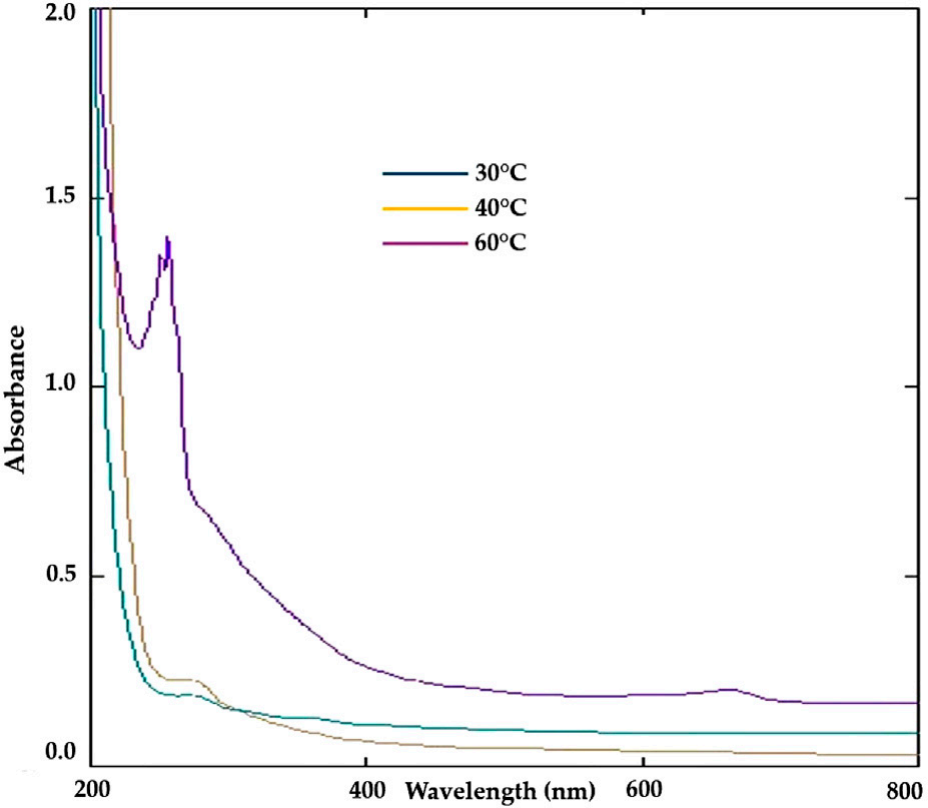
Optimization of temperature for the synthesis of ZnONPs.

**FIGURE 4:**
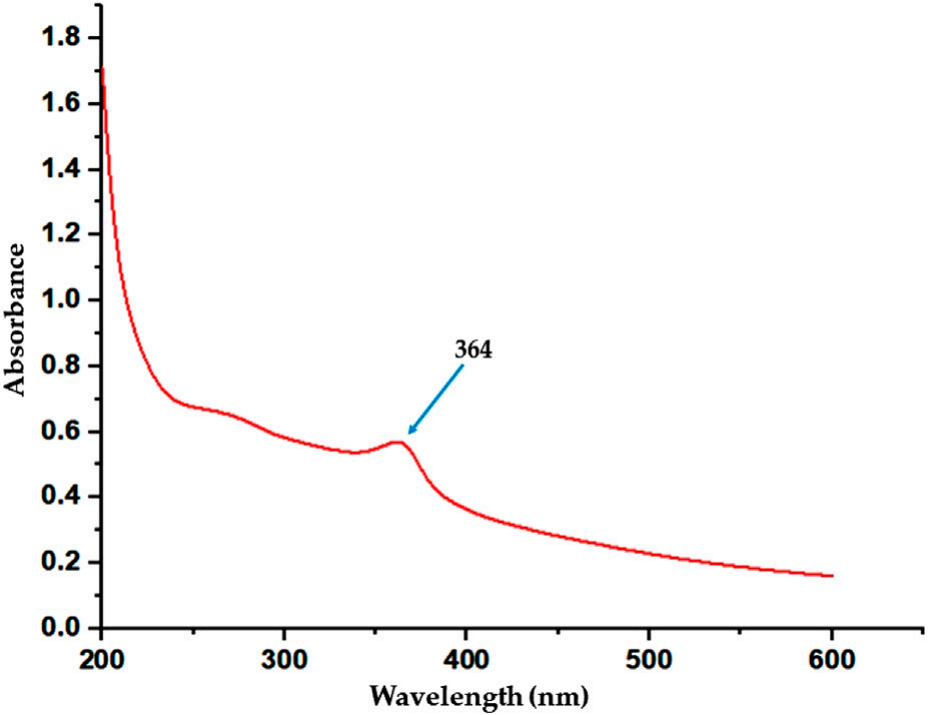
UV–Vis spectra of the synthesized ZnONPs.

The effects of temperature on the synthesis of ZnONPs were verified by examining the UV-Vis spectrophotometry under three different temperature conditions. The spectra showed that the wavelength of the ZnONPs was higher at 40 °C but it dropped to a lower level at 60 °C, which caused smaller zinc nanoparticles to form at higher temperatures, while the size of the zinc nanoparticles increased at higher wavelengths. The absorbance values at different temperatures is shown in Figure 3. The optimum temperature which gave the highest yield of ZnONPs was found to be 50 °C and confirmed by the UV-Vis analysis (Figure 4).

### 3.4 UV-vis analysis

The formation of ZnONPs was detected using ultraviolet-visible spectroscopy. The absorption of the reaction solution was monitored at wavelengths of 200–800 nm using UV-4000 spectrometer to observe the reduction in zinc ions. At 364 nm, the ZnONPs exhibited a surface plasma resonance peak that validated and stabilized the ZnONPs, as shown in Figure 4. The nanoparticle formation can be confirmed efficiently using UV-visible spectroscopy (Anandalakshmi et al., 2016).

### 3.5 FTIR analysis

**FIGURE 5:**
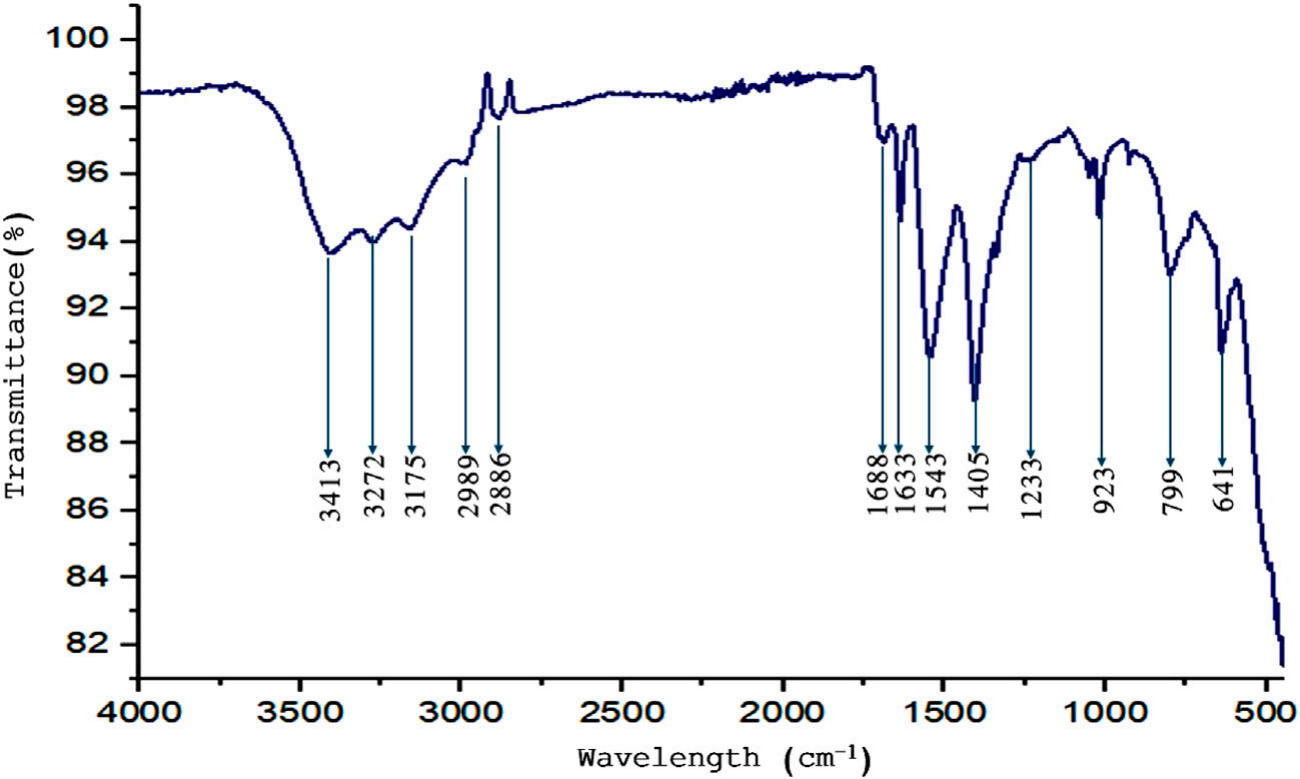
FTIR analysis of the synthesized ZnONPs.

The bioactive components of the aqueous seed extract of C. Revoluta were investigated using FTIR to assess their function in ZnONP production. IR spectroscopy has also been utilized to detect biomolecules in the natural product domain. The diverse IR bands suggest that biomolecules with various functional groups are adsorbed on the surface of ZnONPs (Figure 5). In the current study, the FTIR data is discussed at optimum temperature of 50 °C and pH 10. The peak at 3,414 cm^−1^ is caused by alcohols and phenols stretching their OH groups. The ZnO signal at 469 cm^−1^ may be caused by bonding vibrations between zinc and oxygen. Peaks at 2,854 cm−1 correspond to C≡C and C-H “stretching vibrations,” while peak at 1,405 cm−1 represents “C=C.” C = C. In addition to antioxidants, the seed extract has high levels of phenolic chemicals, flavonoids, and amines. These chemicals are responsible for the creation and stability of the ZnONPs.

### 3.6 SEM with EDX analysis

**FIGURE 6:**
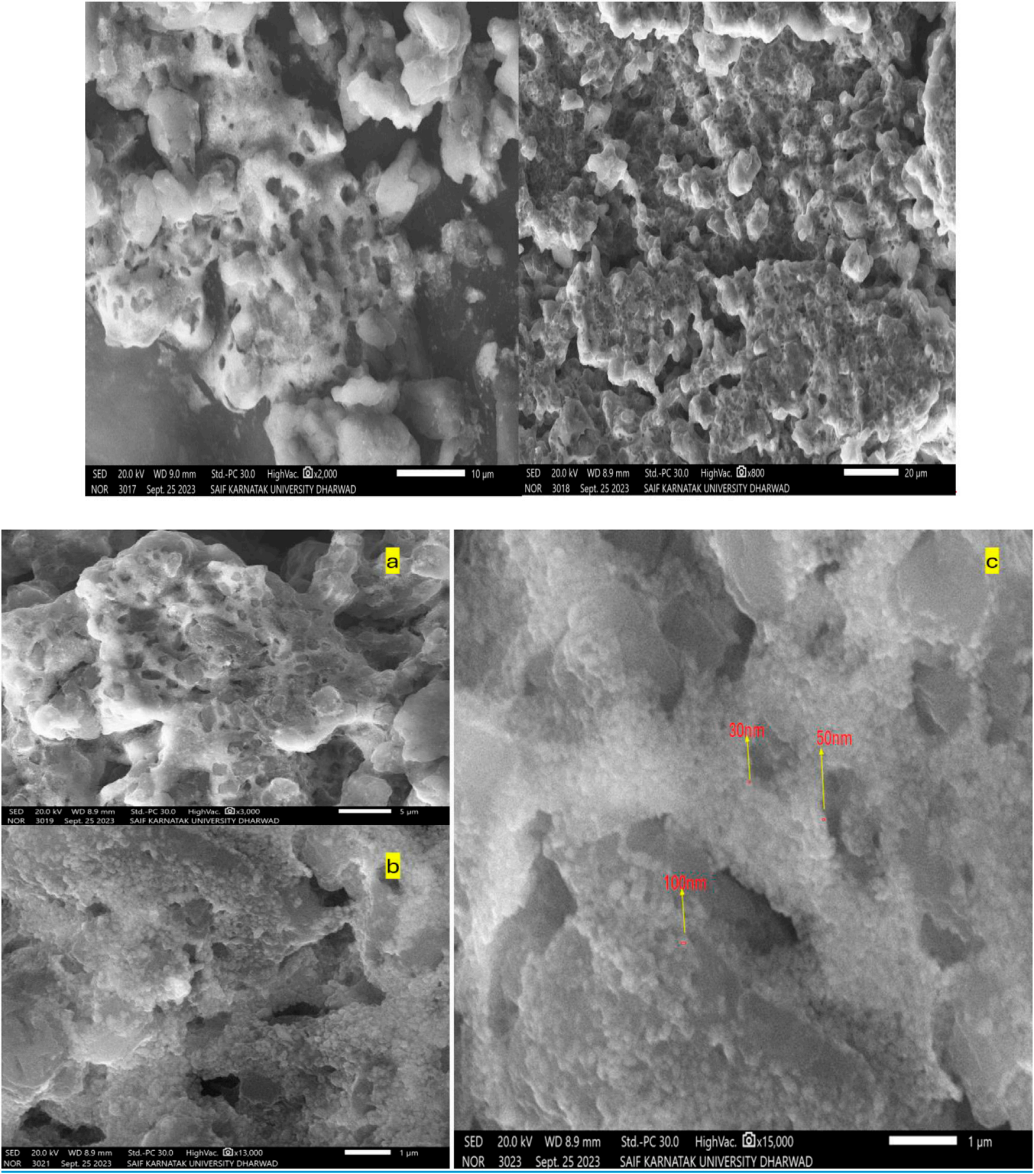
SEM images of the synthesized ZnONPs at increasing magnifications **(a)** ×3,000, **(b)** 130,00X, and **(c)** 150,00X.

**FIGURE 7:**
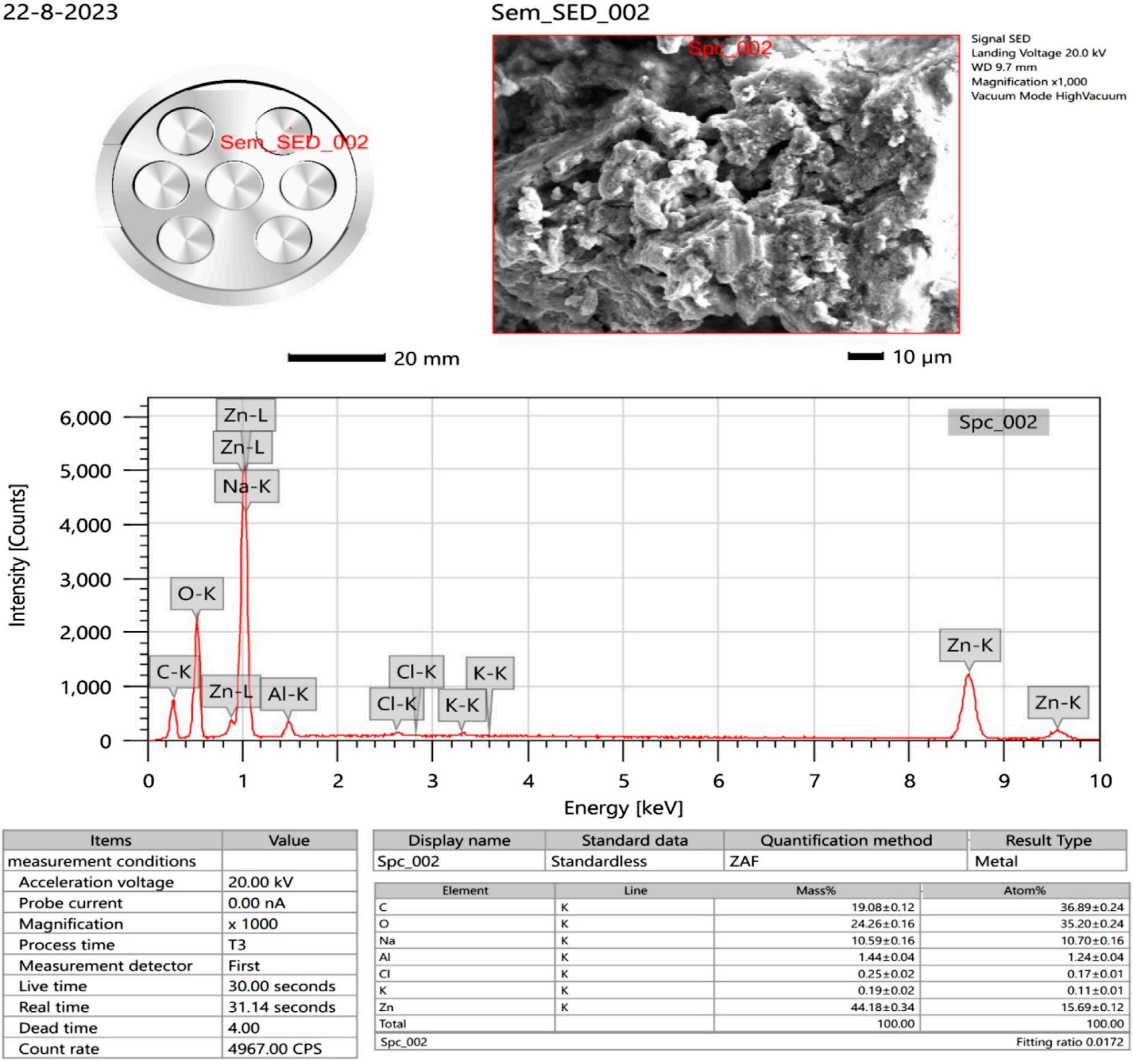
EDX analysis of the synthesized ZnONPs.

The surface morphology of the ZnONPs was investigated using SEM. SEM images captured at increasing magnifications revealed predominantly spherical ZnONPs with notable agglomeration. At magnifications of ×13,000 and 150,00X, and a scale of 1 μm, the nanoparticles were sufficiently resolved to allow approximate size estimation (Figure 6). The average particle size was determined to be ∼30 nm, based on direct measurement from the high-resolution SEM images. The ZnONPs confirmation can be observed in the EDX figure, which displays a strong peak (Figure 7). Energy-dispersive X-ray analysis revealed that Zn and oxygen were the elemental elements. The signals for carbon and oxygen may have originated from bioactive molecules that stabilized the ZnONPs surface, as depicted in Figure 7.

### 3.7 Transmission electron microscopy (TEM) analysis

**FIGURE 8:**
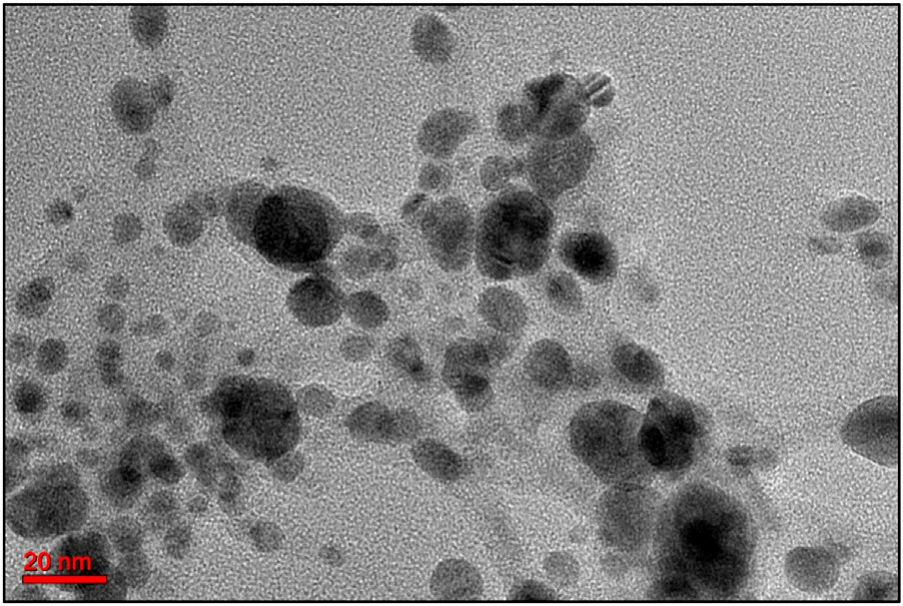
TEM micrographs showing the morphology and particle size of the ZnONPs.

TEM analysis provided detailed insights into the morphological characteristics, particle size, and structural features of the green-synthesized ZnONPs. The micrographs revealed that the nanoparticles were predominantly spherical in shape, consistent with observations from previous SEM analysis. The majority of the particles exhibited sizes ranging between 20 and 60 nm, indicating a relatively uniform size distribution (Figure 8). A notable contrast in electron density was observed, where the edges of the nanoparticles appeared lighter compared to their centers. This variation suggests the presence of organic capping agents, likely biomolecules such as flavonoids, adsorbed onto the surface of the ZnONPs during the green synthesis process. These biomolecules may play a crucial role in stabilizing the nanoparticles, preventing agglomeration, and enhancing their biomedical activity (Zangeneh et al., 2019; Labulo et al., 2022). Overall, the TEM findings confirm the successful synthesis of well-dispersed, spherical ZnONPs with biomolecular surface modifications, which could influence their physicochemical properties and potential applications.

### 3.8 X-ray diffraction (XRD) analysis

**FIGURE 9:**
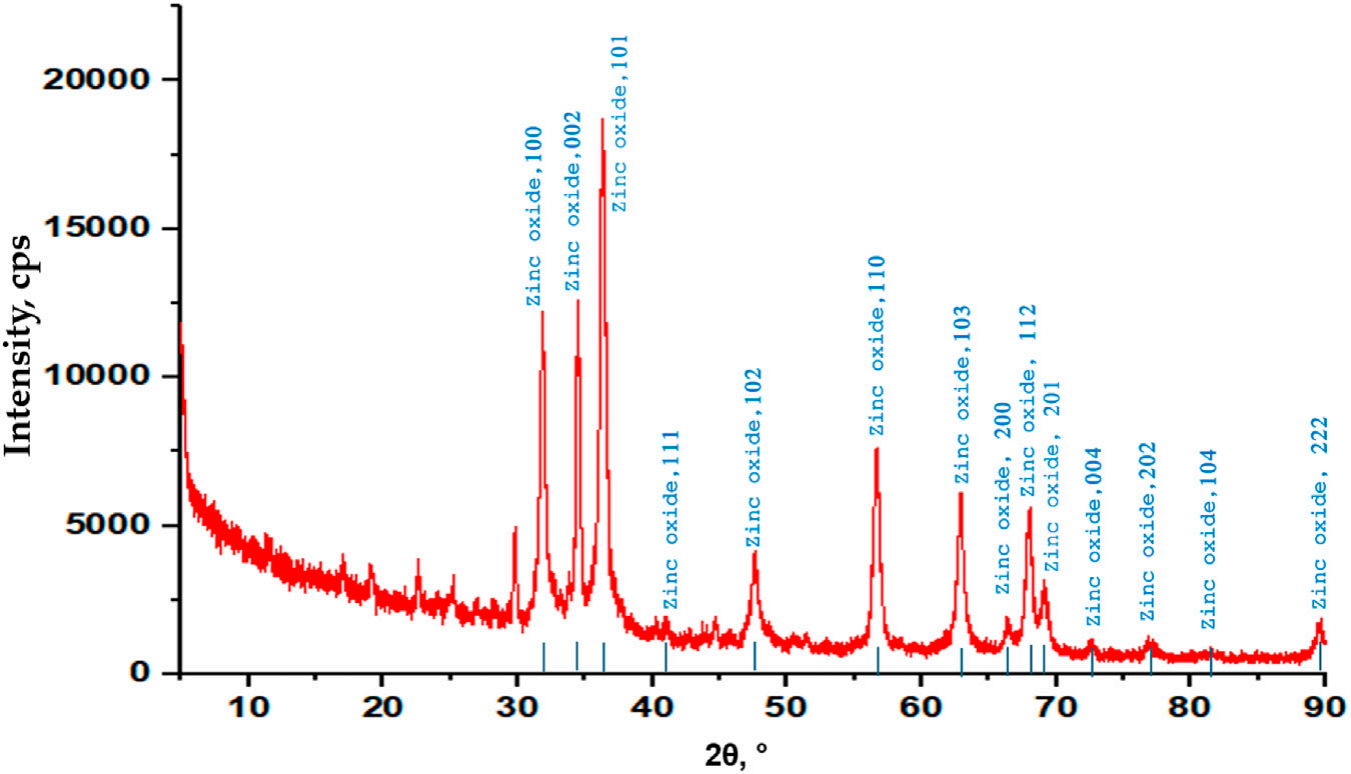
XRD analysis pattern of the synthesized ZnONPs.

The crystalline nature of the ZnONPs was verified by the XRD spectra, and the pattern is shown in Figure 9. The crystalline lattice of ZnONPs exhibits diffraction bands associated with face-centered cubic (FCC) symmetry at 31.41(100), 34.43 (002), 36.24 (101), 47.63 (102), 56.67 (110), 62.73 (103), 67.89 (112), and 69.00 (201). The small size of the particle was confirmed by the broad peaks in the XRD pattern, which also shed light on the ways in which nucleation and crystal nuclei formation were influenced by the experimental conditions. The crystalline structures of the synthesized ZnONPs complied with the JCPDS gold standard (file number: 036–1,451).

### 3.9 Antioxidant activity DPPH assay

**FIGURE 10:**
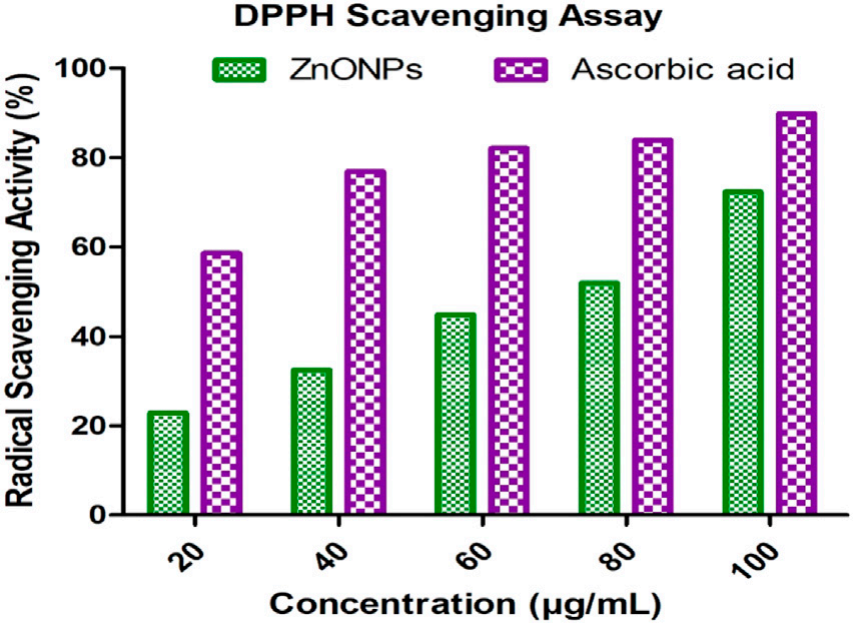
Comparative DPPH scavenging activity of ZnONPs and ascorbic acid.

By comparing the ZnONPs’ antioxidant ability to that that of standard ascorbic acid using the DPPH radical scavenging assay, the percentage radical scavenging activity (RSA) was calculated. The activity of ZnONPs was the highest at 72.36% at 100 μg/mL and the lowest at 22.86% at 20 μg/mL. The standard depicted highest scavenging activity (89.9%) at 100 μg/mL. A comparison is presented in Table 2 and Figure 10.

**TABLE 2:** DPPH assay of ZnONPs and ascorbic acid.

SNo	Concentration (µg/mL)	Ascorbic acid RSA%	ZnONPs RSA%
1	20	58.66	22.86
2	40	76.93	32.52
3	60	82.14	44.84
4	80	83.95	51.88
5	100	89.90	72.36

### 3.10 Antimicrobial activity

**FIGURE 11:**
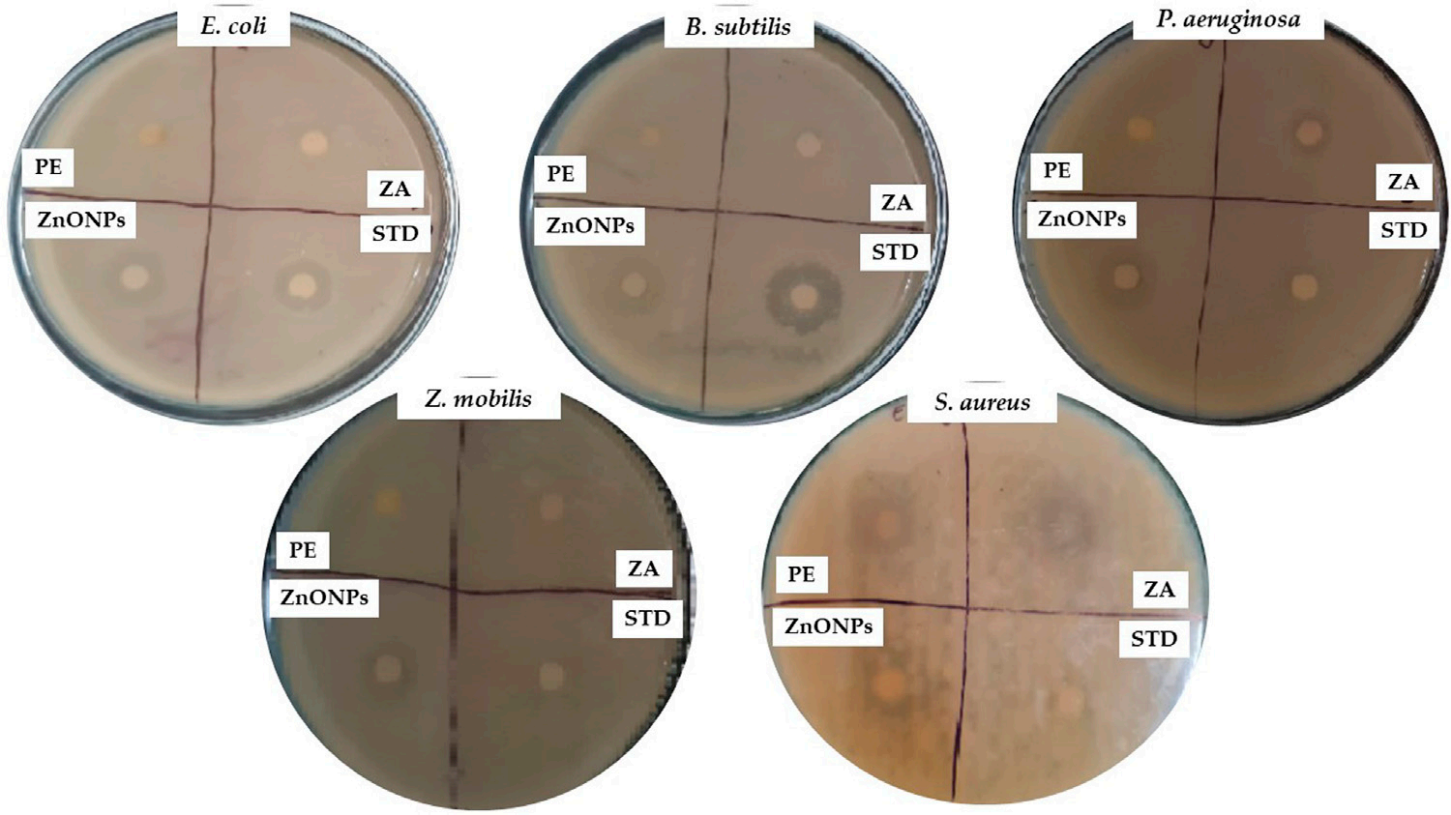
Antibacterial activity of test compounds depicting the zone of inhibition. PE- plant extract; STD-standard drug; ZA- Zinc acetate; ZnONPs- Zinc oxide nanoparticles.

**FIGURE 12:**
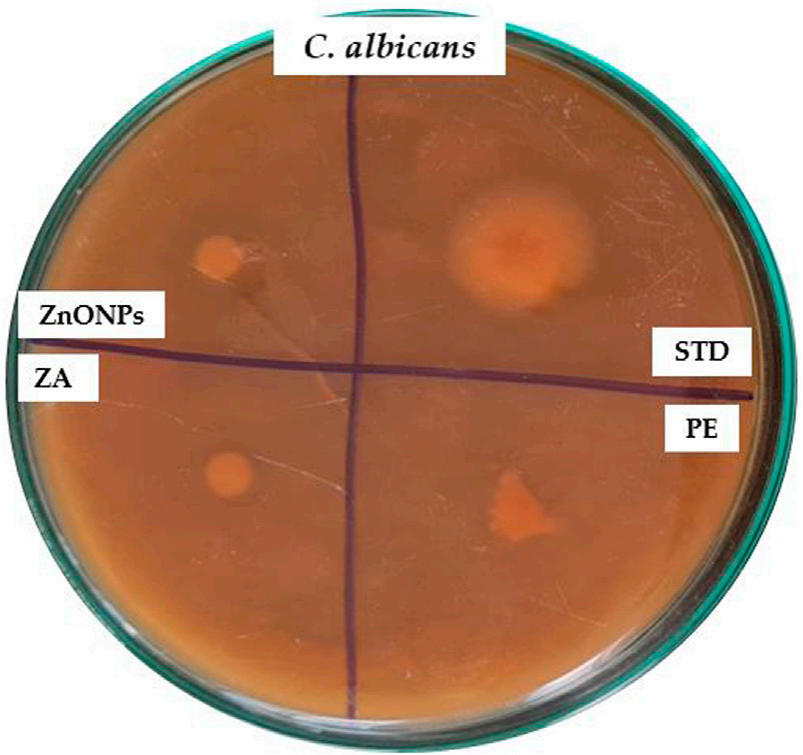
Antifungal activity of test compounds against *C. albicans*. PE- plant extract; STD-standard drug; ZA- Zinc acetate; ZnONPs- Zinc oxide nanoparticles.

ZnONPs demonstrated remarkable inhibitory properties against the microorganisms. The highest levels of inhibition were observed in *P. aeruginosa* (16 mm) and *E*. *coli* (15 mm). The lowest level of inhibition was observed for gram-positive bacteria such as *S. aureus* (11 mm) (Figure 11; Table 3). The difference in inhibition between gram-positive and gram-negative bacteria depends, in part, on the cell wall structure. The Table-3 shows the zones of inhibition for the standard, zinc acetate, and crude extract. The zone of inhibition of the ZnONPs was tested against *C. albicans* (Figure 12).

**TABLE 3:** Antibacterial activity of the synthesized ZnONPs.

Treatment	*E*. *coli (mm)*	*B*. *subtilis (mm)*	*P*. *aeruginosa (mm)*	*Z*. *mobilis (mm)*	*S*. *aureus (mm)*	*C. albicans (mm)*
Zinc acetate	9 ± 0.3	9 ± 0.1	10 ± 0.2	10 ± 0.2	8 ± 0.2	10 ± 0.3
ZnONPs	15 ± 0.5	13 ± 0.3	16 ± 0.3	12 ± 0.2	11 ± 0.1	13 ± 0.1
Plant extract	13 ± 0.2	12 ± 0.1	10 ± 0.1	8 ± 0.1	9 ± 0.1	10 ± 0.1
Standard	15 ± 0.4	16 ± 0.2	15 ± 0.4	9 ± 0.1	20 ± 0.6	25 ± 0.5

These findings are particularly significant given the increasing resistance of wound-associated pathogens to conventional antibiotics. The zones of inhibition observed for ZnONPs, when compared to zinc acetate, crude extract, and the standard (Table 3), underscore the enhanced efficacy of nanoparticulate formulation. Furthermore, the observed antifungal activity against *Candida albicans*, a pathogen often associated with delayed wound healing and superinfection, reinforces the broad-spectrum potential of ZnONPs. Collectively, these antimicrobial properties suggest that ZnONPs could serve as a promising adjunct or alternative in wound management therapies, potentially reducing infection risk and promoting a more favorable healing environment.

### 3.11 Cytotoxic activity against MDAMB-231

The MDAMB-231 cell line was used to investigate ZnONPs’ cytotoxic effect. The standard group consisted of cisplatin, while the control group consisted of untreated MDAMB-231 cells. The findings showed that the percentage of cell viability dropped as the concentration rose. The % of cell viability was found to be 79.57 ± 0.003 at an initial concentration of 20 μg/mL and to drop to 23.51 ± 0.006 at its maximum concentration of 100 μg/mL. The standard drug cisplatin showed 17.33 ± 0.006 (Table 4). In case of IC_50_ for ZnONPs, it was found to be 58.39 μg/mL.

**TABLE 4:** Percentage of cell viability of MDAMB-231.

SNo	Treatment	Conc. in µg/mL	% Cell viability
1	ZnONPs	20	79.57 ± 0.003
		40	61.04 ± 0.002
		60	46.08 ± 0.002
		80	34.20 ± 0.004
		100	23.51 ± 0.006
2	Cisplatin	15	17.33 ± 0.006
IC_50_ of ZnONPs = 58.39 μg/mL			

**FIGURE 13:**
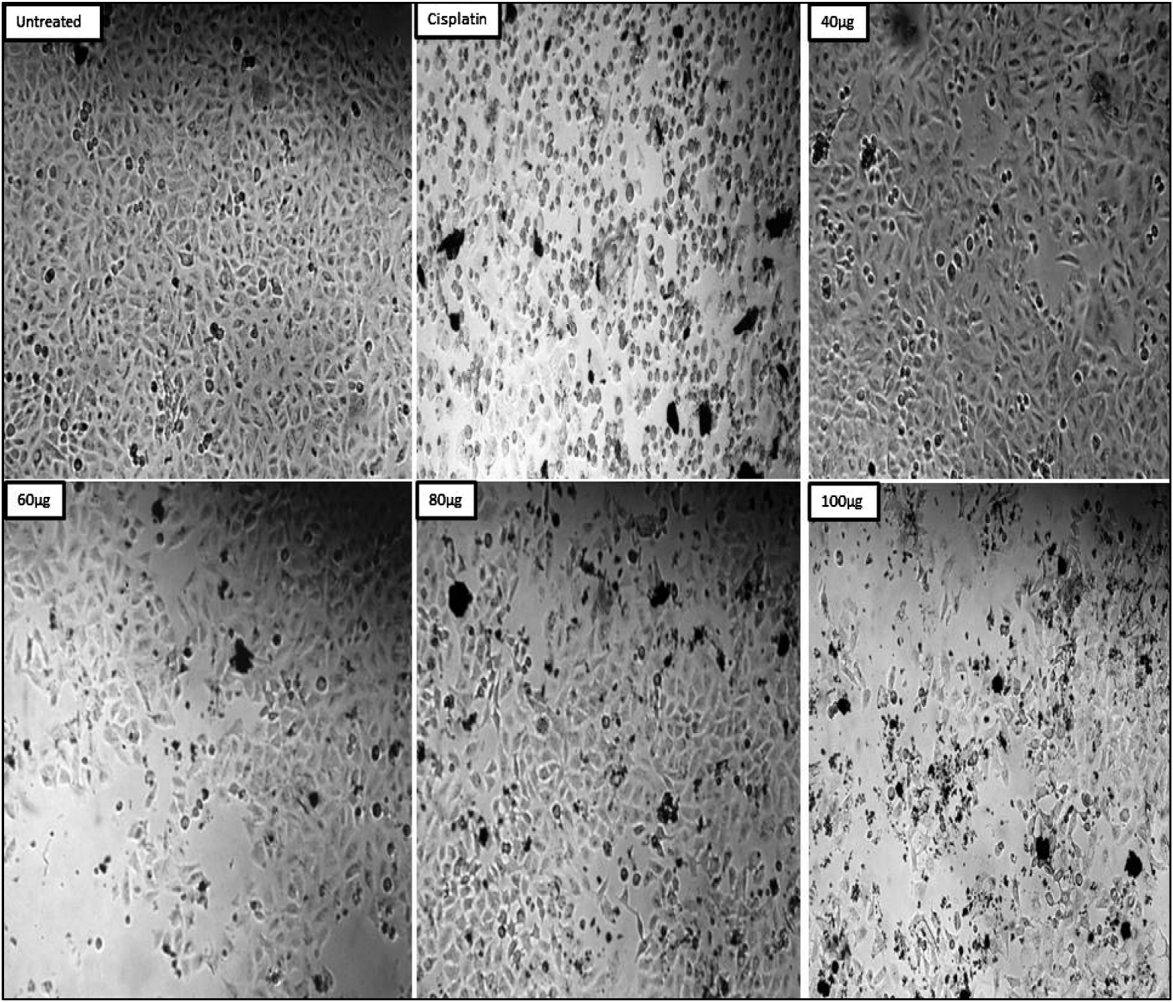
Morphological changes observed on MDAMB-231 cells post treatment with ZnONPs.

The morphological microscopic study indicated that the untreated cells in the test sample exhibited morphological features different from those of ZnONPs-treated cells. ZnONPs-treated cells showed the formation of apoptotic bodies, cell expansion, cell shrinkage, and cell turgidity. In contrast, untreated cells appeared healthy, normal-shaped cells without intracellular spaces (Figure 13).

Cancer is characterized by a progressive increase in the number of cell divisions. The prevalence of this condition is a cause of concern as it leads to substantial illness and death, posing a significant global health challenge. Moreover, there is a growing need for effective treatments to combat cancer (Anand et al., 2023; Burguin et al., 2021). The TNBC cell line is commonly used in late-stage breast cancer research.

Tackling cancer is a formidable challenge, especially effective treatments for rapidly growing tumors. Chemotherapy, which is effective in treating cancer, has limitations due to its low specificity and dose-limiting toxicity. The discovery of effective therapies and drugs for the treatment of different types of cancers poses a significant challenge. Therefore, traditional approaches require the use of controlled-release technology and targeted drug delivery, resulting in enhanced efficacy and reduced harm. Advancements in the field of nanomaterials have the potential to completely transform this approach to cancer diagnosis and therapy (Patel et al., 2024). They are utilized to visualize tumors in their primary and secondary locations, and can also serve as carriers for anticancer drugs (Nune et al., 2009).

### 3.12 Effect of *C. revoluta* ZnONPs on *in vitro* cell migration assay

**FIGURE 14:**
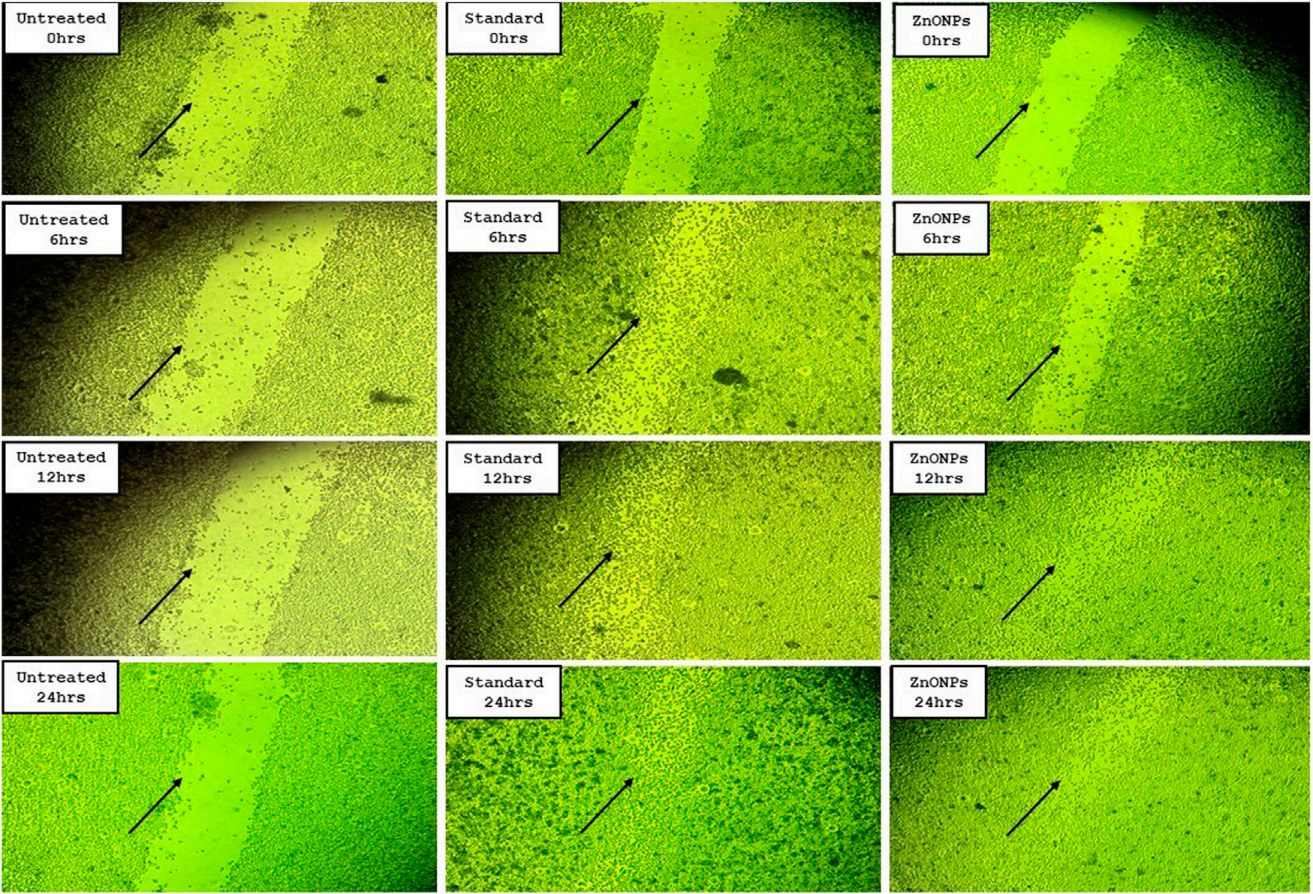
Cell migration assay images for *C. revoluta* ZnONPs depicting the wound closure in the treatment groups.

As per the results, the group treated with ZnONPs had the second-highest cell migration, behind the standard medication ascorbic acid (Table 5). The % of wound closure for ZnONPs was 76.80% after 24 h of incubation, compared to 93.45% for normal ascorbic acid (Figure 14).

**TABLE 5:** Cell migration assay results.

S. No	Test sample	Duration	Cell migration in mm	Percentage wound closure (24 h)
1	Untreated	6 h	3.56	8.12%
		12 h	2.31	
		24 h	1.87	
2	Ascorbic acid	6 h	10.54	93.45%
		12 h	7.32	
		24 h	3.44	
3	Sample	6 h	6.16	76.80%
		12 h	4.10	
		24 h	3.67	

Another avenue for the biomedical application of metal nanoparticles is wound healing and tissue regeneration. Wound healing is a complex and vital series of carefully coordinated biochemical and cellular events that work together to restore the skin integrity. The effectiveness of wound healing in the occlusion of injured tissue greatly depends on the materials used in wound dressings. Extensive studies have been conducted on the effectiveness of traditional wound healing therapies in both experimental and clinical settings. These investigations have yielded a vast amount of valuable insights into the role of these traditional therapies. Cell migration plays a crucial role in the wound-healing process, ultimately determining the effectiveness of the active agent in promoting complete wound closure (Rodrigues et al., 2019). Nanoparticles have shown promise in addressing wound infections, owing to their distinct characteristics. Consequently, scientists have been exploring alternative therapeutic methods that utilize environmentally friendly options such as medicinal plants. The synthesis of nanoparticles derived from plants is characterized by stability, rapidity, and cost-effectiveness. Utilizing nanotechnology in diagnostics and treatment provides a promising avenue for addressing the intricate nature of the normal wound-healing process, the specific types of cells involved, and the underlying causes of chronic wounds. From a conceptual standpoint, the use of nanotherapeutics in cutaneous wound healing offers significant benefits.

### 3.13 Effect of *C. revoluta* ZnONPs on *in vivo* wound healing

**FIGURE 15:**
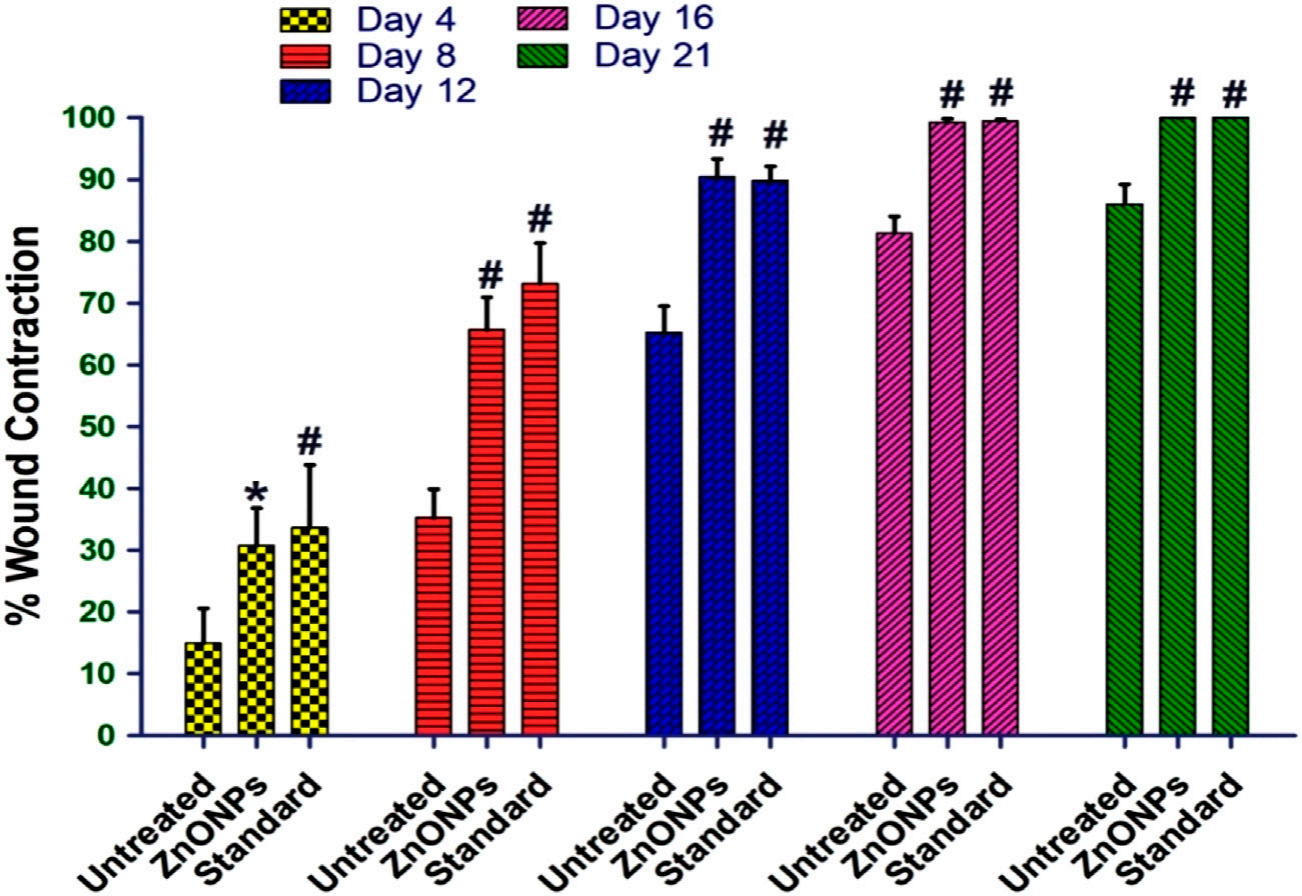
The percentage wound closure in the *in vivo* model. The treatment groups showed statistical significance *p < 0.01; #p < 0.001compared to untreated (control) group.

**FIGURE 16:**
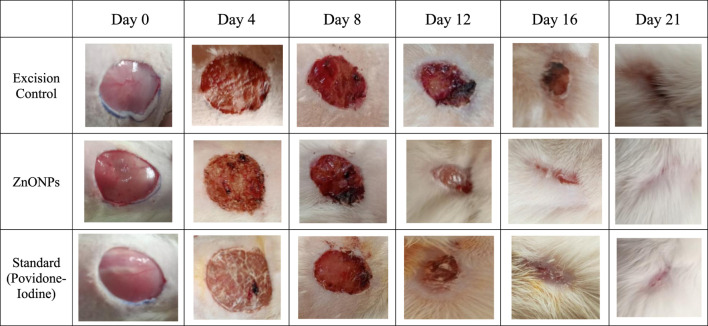
Images of excision wound model at various time intervals.

The *in vivo* excision wound healing model in rats further supported the encouraging *in vitro* wound healing outcomes. Table 6 and Figure 15 show the percentages of *C. revoluta* ZnONPs and povidone-iodine that cause wound contraction *in vivo*. By day 21, the animals’ ability to heal wounds had significantly improved after receiving ZnONPs and povidone-iodine. It required 16.4 days for the animals in the control group to epithelize, whereas 12.2 and 11.5 days for the animals treated with ZnONPs and povidone-iodine, respectively, were significantly shorter. In comparison to the control group, which received no treatment, the groups that got ZnONPs and Povidone-Iodine exhibited a markedly faster rate of full epithelization (p < 0.01). The ZnONPs ointment had a similar healing effect to the povidone-iodine ointment, as seen in Figure 16. When ZnONPs are applied topically, they demonstrate a remarkable capacity to accelerate wound healing and promote tissue regeneration. These findings indicate that the use of nanoparticles has a profound impact on wound healing. These effects can be attributed to stimulation of blood vessel formation, enhanced collagen production, and accelerated regeneration of the outer layer of the skin.

**TABLE 6:** Effect of *C. revoluta* ZnONPs in excision wound model.

Groups		Wound diameter (mm)					Wound contraction (%)					
	D0	D4	D8	D12	D16	D21	D0	D4	D8	D12	D16	D21
Excision (Control)	25.5± 0.577	23.5± 0.577	20.5± 0.5	15± 0.816	11± 0.816	9.5± 1.290	0	14.93± 5.68	35.25± 4.67	65.27± 4.25	81.29± 2.76	86.02± 3.21
*C. revoluta* ZnONPs	25.25± 0.5	21± 0.816	14.75± 0.829	7.75± 1.258	2± 0.81	0.25± 0.5	0	30.69± 6.12*	65.67± 5.29^#^	90.39± 2.96^#^	99.29± 0.53^#^	99.96± 0.08^#^
STD (Povidone-Iodine)	26± 0.5	20.5± 1.290	13± 1.224	8± 0.816	1.75± 0.5	0	0	33.64± 10.22^#^	73.15± 6.55^#^	89.82± 2.33^#^	99.49± 0.23^#^	100 ± 0.0^#^

The treatment groups showed statistical significance, *p < 0.01; #p < 0.001 compared to untreated (control) group.

Wound healing is a multifactorial process influenced not only by hormonal differences but also by age, genetic background, immune status, and environmental factors. In this study, both male and female Wistar rats were included to account for potential sex-dependent differences in response to treatment with ZnONPs and provides a more comprehensive understanding of their therapeutic efficacy. While hormonal influences, such as estrogen’s role in modulating inflammation and promoting tissue regeneration, are well documented, the current study’s primary objective was to evaluate the overall wound healing capacity of the biosynthesized ZnONPs across a representative sample. Future studies could stratify the animals based on sex and investigate hormonal contributions more specifically to isolate mechanistic differences.

### 3.14 Hydroxyproline content

**FIGURE 17:**
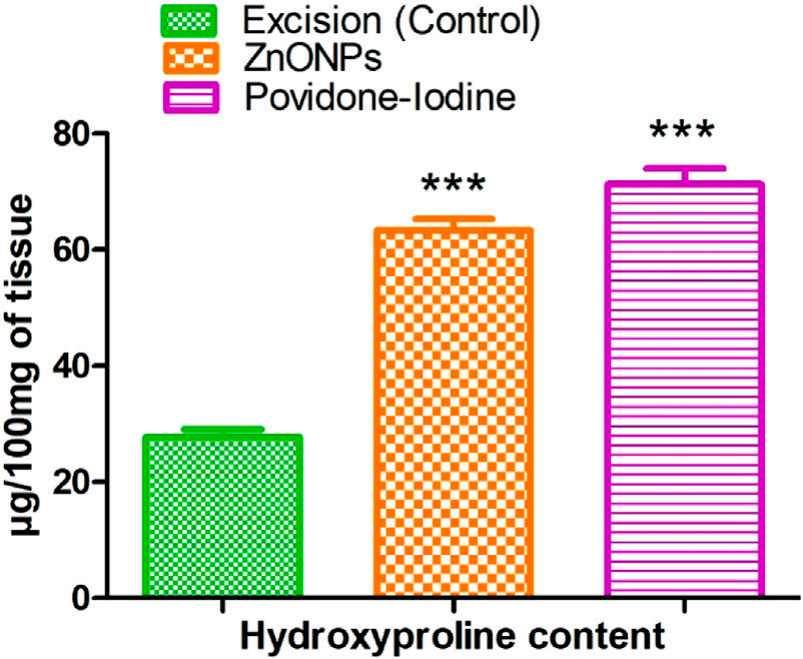
Effect of ZnONPs on hydroxyproline content. The treatment groups showed statistical significance *** <0.001 compared to untreated (control) group.

The effect of *C. revoluta* NPs on the amount of hydroxyproline in the regenerated tissue is shown in Figure 17. The excision control animals had a hydroxyproline level of 27.77 ± 1.31. Animals treated with ZnONPs had a considerably increased hydroxyproline level (63.40 ± 1.88). Hydroxyproline is an amino acid that is a major component of collagen, which is the primary structural protein in the extracellular matrix of connective tissues. An increase in hydroxyproline levels is generally associated with enhanced collagen synthesis and deposition, which is an important aspect of wound healing and tissue regeneration. The results suggest that the *C. revoluta* ZnONPs and povidone-iodine treatments were able to stimulate collagen production and deposition in the regenerating tissue, potentially accelerating the wound healing process. The fact that both treatments led to a statistically significant (p < 0.001) increase in hydroxyproline levels compared to the control group highlights the potential therapeutic benefits of these interventions.

## 4 Conclusion

The present study successfully demonstrated synthesis and comprehensive characterization of ZnONPs using the seed extract of *C. revoluta*, a readily available and sustainable source. The ZnONPs exhibited remarkable antimicrobial and antifungal activity, highlighting their potential therapeutic applications against a wide range of microbial pathogens. Furthermore, ZnONPs demonstrated excellent cytotoxic activity against the highly aggressive triple-negative breast cancer cell line MDAMB-231. The ZnONPs also displayed significant wound-healing properties and enhanced cell migration, indicating their potential for wound management and tissue regeneration applications. Additionally, ZnONPs possessed potent antioxidant activity, as confirmed by DPPH assays, which could contribute to their overall therapeutic efficacy. Further in-depth investigations and clinical evaluations are warranted to fully explore the therapeutic potential of eco-friendly *C. revoluta* ZnONPs.

### 4.1 Future aspect

Significant progress has been made in the synthesis of ZnONPs; however, the fundamental mechanisms underlying nanoparticle formation, particularly via green synthesis routes, remain incompletely understood. Further research is essential to elucidate these pathways, which would contribute to enhancing the reproducibility, efficiency, and scalability of eco-friendly synthesis processes. Current studies indicate that *C. revoluta* seed extract holds considerable promise as a sustainable and effective medium for the green synthesis of ZnONPs. Its potential applications, especially in the biomedical and pharmaceutical sectors, highlight its value in advancing environmentally friendly nanotechnology.

## Data Availability

The original contributions presented in the study are included in the article/supplementary material, further inquiries can be directed to the corresponding authors.

## Abbreviations

EDS-SEM, Scanning Electron Microscopy and Energy Dispersive X-ray Spectroscopy; XRD, X-ray Diffraction; FTIR, Fourier Transform Infrared Spectroscopy.
